# Supplementary material for: The mTOR Inhibitor Rapamycin Counteracts Follicle Activation Induced by Ovarian Cryopreservation in Murine Transplantation Models
Source: Medicina (Kaunas). 2023 Aug 16;59(8):1474. doi: 10.3390/medicina59081474 (PMC10456585; doi:10.3390/medicina59081474)
Supplement: Supplementary file 1 [file medicina-59-01474-s001.zip › medicina-2498732-supplementary.pdf]

Supplementary

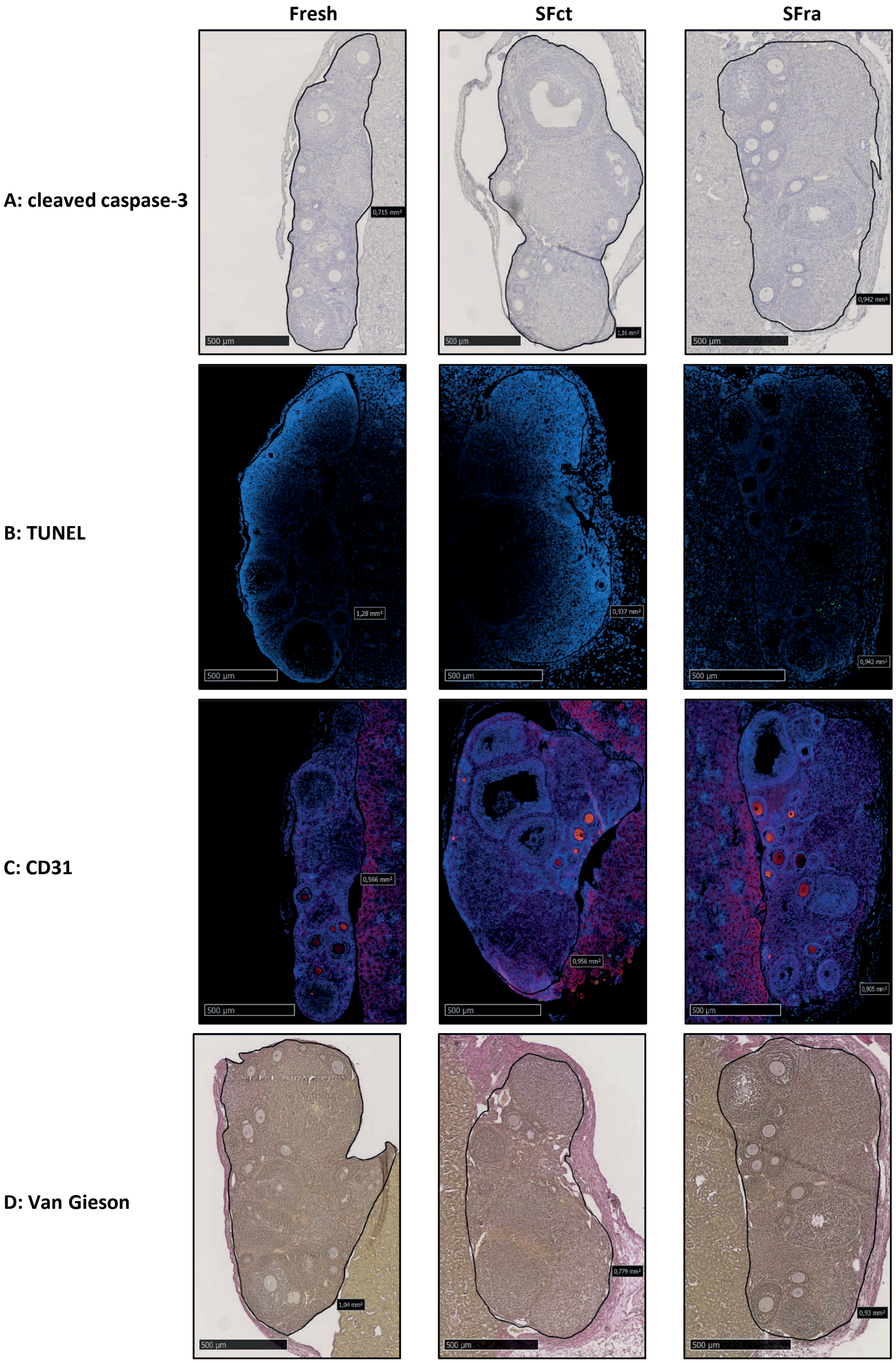

Figure S1. Effects of adding rapamycin to the freezing medium on apoptosis, vascular endothelial cells and fibrosis in mice ovaries fresh, slow-frozen (SF) control (SFct) or slow-frozen with rapamycin (SFra), autotransplanted under the kidney capsule of C57Bl/6 mice (4-weeks-old) for three weeks. Representative images of cleaved caspase-3 (**A**), TUNEL (**B**), CD31 (**C**) and Van Gieson (**D**) staining of fresh, SFct or SFra mice ovaries transplanted under the kidney capsule. Red staining = DDX4, green staining = TUNEL or CD31. Van Gieson: nucleus in black, cytoplasm in yellow, collagen and muscle in red. n = 5-8 ovaries per group.

A: cleaved caspase-3

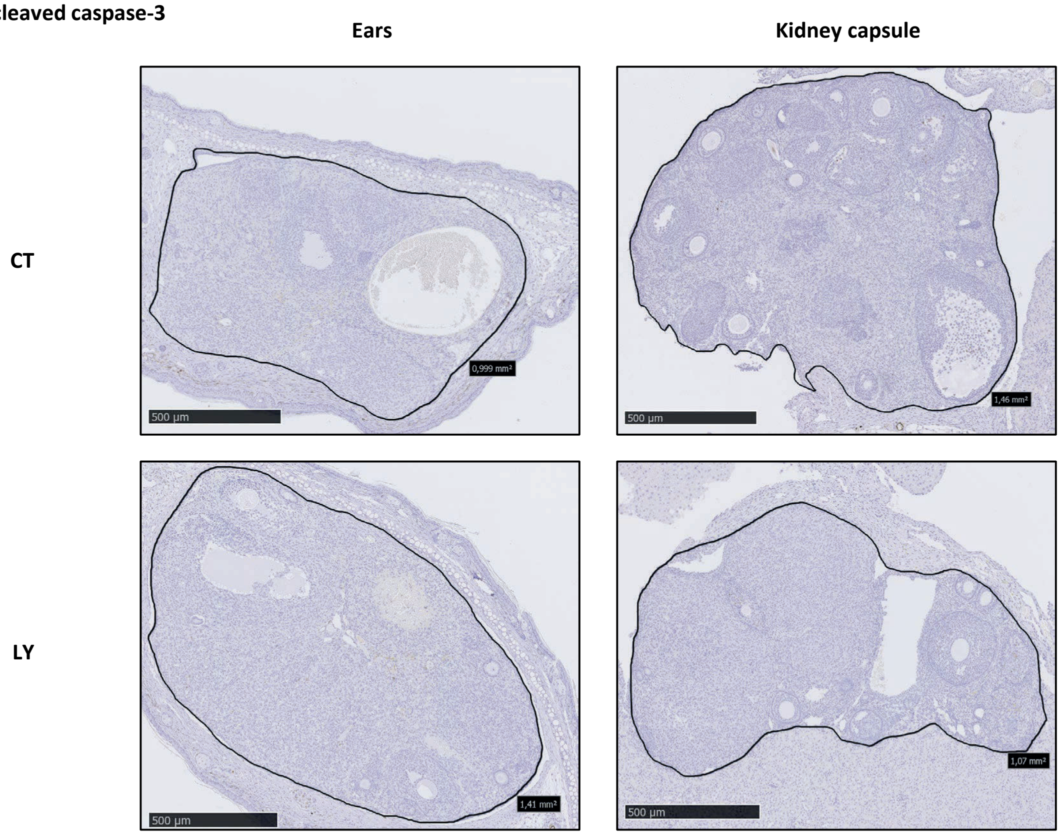

B: TUNEL

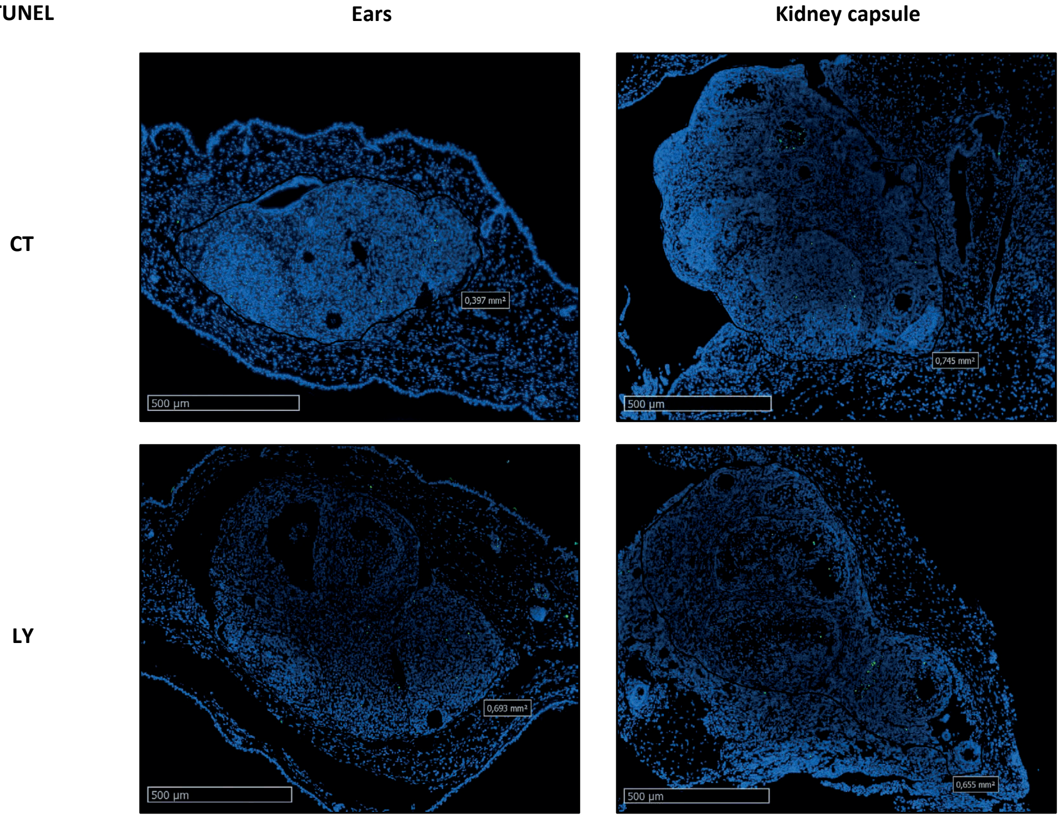

Figure S2. Comparison of apoptosis for LY294002 (LY) injection after ovarian autotransplantation into C57Bl/6 mice (4-weeks-old) either locally in the ears or IP when ovaries were transplanted under the kidney capsule. Representative images of cleaved caspase-3 (**A**) and TUNEL (**B**) staining of fresh mice ovaries transplanted either between the skin layers of the ears or under the kidney capsule, followed by local injection with LY or vehicle control for ovaries transplanted between skin layers of the ears, or intraperitoneal (IP) for ovaries transplanted under the kidney capsule. Green staining = TUNEL. n = 6-8 ovaries per group. Ears = transplantation of ovaries between skin layers of ears followed by local LY/control injection, Kidney capsule = transplantation of ovaries under the kidney capsule followed by IP LY/control injection, CT = control injection, LY = injection with LY.
